# Supplementary material for: Value-based attentional capture affects multi-alternative decision making
Source: eLife. 2018 Nov 5;7:e39659. doi: 10.7554/eLife.39659 (PMC6218187; doi:10.7554/eLife.39659)
Supplement: Supplementary file 1. [file elife-39659-supp1.docx]

**Supplementary File 1 – Tables for statistical results and model parameters**

**Table S1.** Comparison of overall performance with the Chau2014 data.

| Mean | | | | | | |
| --- | --- | --- | --- | --- | --- | --- |
|  | Chau2014 | Experiment 1 | Experiment 2 HP | Experiment 3 | Experiment 4 | Combined |
| Relative; all trials | .715 | .688 (.124) | .709 (.721) | .712 (.904) | .713 (.938) | .706 (.543) |
| Absolute; all trials | .667 | .628 (.072^+^) | .651 (.406) | .661 (.775) | .645 (.250) | .645 (.199) |
| Relative; D absent trials | .727 | .703 (.180) | .726 (.971) | .726 (.958) | .732 (.783) | .722 (.768) |
| Absolute; D absent trials | .699 | .668 (.132) | .690 (.636) | .697 (.910) | .694 (.773) | .687 (.471) |
| Relative; D present trials | .701 | .670 (.147) | .689 (.552) | .698 (.848) | .693 (.608) | .687 (.398) |
| Absolute; D present trials | .635 | .587 (.091^+^) | .611 (.330) | .626 (.700) | .596 (.080^+^) | .602 (.129) |
| Standard Deviation | | | | | | |
| Relative; all trials | .052 | .066 (.267) | .062 (.440) | .067 (.266) | .059 (.533) | .063 (.320) |
| Absolute; all trials | .065 | .082 (.267) | .067 (.865) | .069 (.788) | .074 (.517) | .074 (.499) |
| Relative; D absent trials | .062 | .062 (.970) | .063 (.919) | .076 (.365) | .067 (.731) | .067 (.719) |
| Absolute; D absent trials | .072 | .073 (.970) | .059 (.365) | .077 (.780) | .076 (.826) | .072 (.950) |
| Relative; D present trials | .058 | .087 (.060^+^) | .075 (.232) | .067 (.501) | .062 (.752) | .073 (.240) |
| Absolute; D present trials | .074 | .111 (.060^+^) | .087 (.458) | .081 (.664) | .084 (.518) | .092 (.260) |

*Note.* “Relative” refers to relative choice accuracy; “Absolute” refers to absolute choice accuracy. Values in parentheses refer to *p*-values of two-sample *t*-tests / two-sample *F*-tests for comparing means / variances between the Chau2014 dataset and our datasets. **^+^***p* < .1.

**Table S2.** Regression analyses of relative choice accuracy.

|  | Exp. 1 | Exp. 2 HP | Exp. 3 | Exp. 4 | All (exc. 2 LP) | Exp. 2 LP |
| --- | --- | --- | --- | --- | --- | --- |
| HV-LV | **0.432***** | **0.492***** | **0.453***** | **0.555***** | **0.492***** | **0.760***** |
| HV+LV | **-0.098*** | **-0.171**** | **-0.171**** | **-0.154***** | **-0.146***** | **-0.210***** |
| HV-D | 0.041 | 0.017 | 0.031 | -0.006 | 0.017 | 0.086 |
| (HV-LV)×(HV-D) | 0.096^+^ | -0.046 | **0.082**** | 0.038^+^ | **0.031*** | **0.092*** |
| D present | **-0.157*** | **-0.166*** | **-0.139*** | **-0.208***** | **-0.174***** | -0.016 |

*Note.* Values represent average regression coefficients (intercepts omitted).

**^+^***p* < .1, **p* < .05, ***p* < .01, ****p* < .001

**Table S3.** Regression analyses of absolute choice accuracy.

|  | Exp. 1 | Exp. 2 HP | Exp. 3 | Exp. 4 | All (exc. 2 LP) | Exp. 2 LP |
| --- | --- | --- | --- | --- | --- | --- |
| HV-LV | **0.377***** | **0.399***** | **0.391***** | **0.456***** | **0.412***** | **0.712***** |
| HV+LV | -0.057 | **-0.139**** | **-0.129**** | **-0.093***** | **-0.100***** | **-0.189***** |
| HV-D | **0.164***** | **0.173***** | **0.209**** | **0.150***** | **0.169***** | 0.127^+^ |
| (HV-LV)×(HV-D) | 0.046^+^ | -0.007 | **0.099**** | **0.049*** | **0.046***** | **0.113**** |
| D present | **-0.355***** | **-0.357***** | **-0.323***** | **-0.458***** | **-0.386***** | -0.004 |

*Note.* Values represent average regression coefficients (intercepts omitted).

**^+^***p* < .1, **p* < .05, ***p* < .01, ****p* < .001

**Table S4.** Regression analyses of the influence of the value of D on making specific errors.

|  | Exp. 1 | Exp. 2 HP | Exp. 3 | Exp. 4 | All (exc. 2 LP) |
| --- | --- | --- | --- | --- | --- |
| Choice of D | **0.424*****(2) | **0.410***** (1) | **0.384***** (1) | **0.396*****(2) | **0.403***** (6) |
| Too slow | **0.202*** (1) | **0.212*** (3) | 0.116 (5) | 0.014 (3) | **0.121**** (12) |
| Choice of LV | -0.011 | 0.017 | -0.018 | **-0.059**** | -0.024^+^ |
| Choice of empty quadrant | 0.037 (7) | -0.058 (2) | -0.058 (8) | 0.017 (7) | -0.007 (24) |

*Note.* Each error type is analyzed by a separate logistic regression analysis. Values represent average regression coefficients (intercepts omitted). Values in parentheses show the number of participants that made the respective error never or only once, and were thus excluded from the respective analysis (results of Experiment 2, Group LP, are omitted because of too few errors). There seems to be a trend (*p* < .1) that Ds of higher value lead to less choices of LV. However, this effect is driven by the fact that participants made more of the other errors (i.e., choosing D itself; being too slow) when D had a high value (so that choosing LV must become less likely): Restricting the analysis to trials in which either HV or LV were chosen eliminates the trend (*t*(122) = 0.18, *p* = .861). Thus, there was no violation of IIA.

**^+^***p* < .1, **p* < .05, ***p* < .01, ****p* < .001

**Table S5.** Regression analyses of response times.

|  | Exp. 1 | Exp. 2 HP | Exp. 3 | Exp. 4 | All (exc. 2 LP) | Exp. 2 LP |
| --- | --- | --- | --- | --- | --- | --- |
| HV-LV | **-13.2***** | **-12.8***** | **-12.0***** | **-19.6***** | **-15.2***** | **-87.9***** |
| HV+LV | **-42.8***** | **-41.7***** | **-40.3***** | **-45.4***** | **-43.1***** | **-113.2***** |
| D | **16.8***** | **15.4***** | **9.9**** | **12.5***** | **13.7***** | **73.6***** |
| D present | **129.6***** | **120.1***** | **120.1***** | **139.1***** | **129.3***** | **414.9***** |

*Note.* Only choices of HV and LV are included in the analyses. Values represent average regression coefficients (intercepts omitted).

***p* < .01, ****p* < .001

**Table S6.** ANOVAs of relative choice accuracy in the novel trials.

|  | Exp. 1 | Exp. 2 HP | Exp. 3 | All (exc. 2 LP) | Exp. 2 LP |
| --- | --- | --- | --- | --- | --- |
| Dominance | **5.85*** | 0.25 | 0.00 | 1.15 | 0.03 |
| Similarity | 0.02 | 2.45 | 0.03 | 0.86 | **7.62*** |
| Interaction | 0.01 | 0.52 | 1.03 | 1.11 | 0.01 |

*Note.* Values represent the *F*-values of the respective factors. “Interaction” refers to the interaction between the two main factors Dominance and Similarity. Experiment 4 did not contain novel trials.

**p* < .05

**Table S7.** ANOVAs of absolute choice accuracy in the novel trials.

|  | Exp. 1 | Exp. 2 HP | Exp. 3 | All (exc. 2 LP) | Exp. 2 LP |
| --- | --- | --- | --- | --- | --- |
| Dominance | **18.89***** | 1.29 | **5.17*** | **17.73***** | 1.12 |
| Similarity | 0.01 | 1.42 | 0.12 | 0.41 | **9.20**** |
| Interaction | 0.09 | 1.54 | 0.56 | 1.70 | 0.05 |

*Note.* Values represent the *F*-values of the respective factors. “Interaction” refers to the interaction between the two main factors Dominance and Similarity. Experiment 4 did not contain novel trials.

**p* < .05, ***p* < .01, ****p* < .001

**Table S8.** Estimated parameter values of MIVAC.

|  | σ | γ | β | π |
| --- | --- | --- | --- | --- |
| *M* | 17.67 | 1.38 | 11.83 | .56 |
| *Md* | 17.43 | 1.41 | 11.99 | .51 |
| *SD* | 9.16 | 0.67 | 4.20 | .29 |
| Min | 5 | -0.88 | -2.09 | .001 |
| Max | 49.83 | 2.98 | 18.90 | 1 |

*Note.* σ = standard deviation of accumulation, γ = value-based attentional capture, β = attention-based enhancement of accumulation, π = probability to identify D as being unavailable.
